# The Matron's Department

**Published:** 1905-09-30

**Authors:** 


### THE MATRON'S DEPARTMENT.

### VII.—TRAINING THE NURSE.

It is the duty of every matron to regard the period of training allotted to her probationers, in each case, as a whole. It is quite possible, under an easy-going system, for two nurses trained simultaneously at the same hospital, to exhibit widely different degrees of efficiency, and this mainly owing to the degree of opportunity afforded in each case. It is now usual to allot at least three years to the training of the nurse, and during that time she should be exercised in medical and surgical nursing in both male and female wards, in night nursing, in the theatre, and in the out-patient and receiving-rooms. She should also be granted experience in any special branch of nursing which may be afforded in her training school. This may be regarded as the bare framework, but to make sure that nurses in training have their time distributed fairly on these lines, demands some considerable amount of skill and forethought on the part of the matron. To this end it is essential to keep a record of each probationer which shall state month by month in what wards her time has been spent, with a note of the report on her work supplied by the sister who has supervised it. In this way only can the matron follow every step of the probationer's career. She can balance the unsatisfactory opinion of one sister against the warm eulogy of another, such contradictory judgments frequently occurring in the case of "tempersome" women, and can watch the evolution of the nurse from the undisciplined novice, without being discouraged at occasional failures. She will be able, by aid of the information thus available, to give a fair opportunity to every probationer of learning every branch of her work. She will avoid the temptation to keep probationers too long in one department of work—the clever because they are useful, and the dull because it is less trouble than teaching them something new, and by a wise distribution of the time which is to be spent in the institution will lay the basis of a sound nursing education.

There are three main branches in the training of the probationer, the practice of nursing, the theory of nursing, and the ethics of nursing. The matron in a good training school is alive to the importance of all three, and has her own methods of providing that no one of them is neglected. We are concerned here not so much with the actual details which go to make up good training, as with the matron's responsibilities.

The practice of nursing is learnt in the daily routine of the wards, and is all too commonly left for the probationer to pick up as she goes along, from the grudging and impatient instruction vouchsafed by her seniors, aided by such wits and powers of observation as nature lends to the task. Irksome as the process often proves, it is at least a sure one, if sufficient time be devoted to it. It is tolerably certain that no woman of ordinary intelligence passes three years in the wards of a good hospital without becoming familiar with all the duties she is expected to perform as a nurse. But should the matron rest satisfied with this? We think not. It is her duty to see that every probationer receives instruction from the very first as her due, and not as a disagreeable concession to her incapacity. It should be made clearly evident that the time spent in each ward is expected to count for something, and to this end the matron should define with her sisters the particular duties in which their successive probationers ought to become proficient under them, and as we have said should receive from them reports at the end. The order in which the probationer's nursing duties are acquired will depend of course on the ward to which she happens to be assigned, but it is for the matron to arouse in every ward a spirit of willing and skilful instruction which shall reduce the period of incapacity in the newcomer to a minimum and turn her speedily from an unhappy blunderer to a competent assistant. It seems rather thankless and dreary work teaching a succession of pupils to perform elementary duties. It can be stimulated till it becomes a pleasure if there is some one at the head of affairs who takes a keen interest in it, appreciates its difficulties, and finds time to watch the progress made. A regular system, embracing the elementary duties in which the probationer is expected to become proficient during her first year, should be drawn up by the matron, and it will be all the more successful if she invites the co-operation of her ward sisters in its construction, since it is for them to apportion the work and carry out the plan of practical education in nursing which shall be agreed on. It should be well recognised both by the probationer herself and by those responsible for her instruction that it is due to the institution that the period of inefficiency be reduced to a minimum.

Although it may not fall to the matron to direct ostensibly the theoretical training of the probationers, it is certain that the standard of proficiency attained will depend largely on her. It is important that she herself attend the lectures given by members of the medical staff, and it is for her to provide that the pupils receive after instruction in the same subject, when the lecture can be carefully gone through, and lessons given in note-taking and in answering examination papers. The classes which arise out of the lecture should be graded into two or more divisions, corresponding to the standard of intelligence or education among the pupils, and backward pupils placed under the charge of one of the sisters responsible for coaching them. The usual course of theoretical training afforded to probationer nurses consists in lectures on—(*a*) physiology; (*b*) medical nursing; (*c*) surgical nursing. Some knowledge of physiology is, of course, essential to enable the nurse to obey instructions intelligently. But this elementary knowledge of physiology can, as we have said, be acquired quite as readily before entering the hospital as the arts of reading and writing. Medical and surgical nursing cannot, on the contrary, be studied with full advantage except by nurses actually engaged in ward work; and it is, therefore, of the greatest importance that as much time as possible should be secured for this indispensable part of the nurse's education during the time she spends in hospital. The matron may do much by her own zeal to stimulate the interest of her nurses in the teaching available for them. Lectures which appear stiff or dull to ignorant girls may be filled with interest if they are followed up by a class of keen comment and illustration. And if the matron will train herself to give this class in person she will be well repaid. Her opportunities of coming into personal relations with her probationers are not many, and an occasion for addressing them collectively is one which she and they will grow to value in proportion to her own sense of its importance.

And this leads to the consideration of the third branch of training—the ethics of nursing. Certain persons are found to contend that the matron of a hospital has nothing to do with moulding the character of her nurses, but must confine herself merely to teaching them their work. We are not concerned to refute this contention because, whether she will or no, the head of a training school must inevitably influence her nurses for good or for evil. Things which she considers of importance, whether it be the punctilious assertion of her own dignity, or the smart appearance of the wards, or the repression of tittle-tattle, will be the things which will be brought about; if she is unpunctual, the nurses will be unpunctual; and if she allows herself to gossip, gossip will be the order of the day. But, apart from this general inevitable influence exercised by the head of a household on her subordinates, there is a special code of ethics binding on the nurse in her professional character, and many of the temptations attaching to the practice of private nursing would be avoided if probationers were well grounded in these obligations during their time of training. The minor obligations relating to dress and manners; the greater obligations of reticence and of loyalty to the medical officer in charge of the case; these seem simple enough—almost too obvious—to the outsider. But in practice the duties of the nurse towards herself, the patient, the patients' friends, and the doctor are full of complexity. It is quite impossible to give lectures on nurses' difficulties. But the matron whose eyes are open to such difficulties may constantly refer to and discuss them with her probationers in her weekly class on nursing. In a purely business-like way, without the slightest attempt at preaching, she may embue her subordinates with a sense of their high calling. And, alive to the fact that many nurses with high ideals fail in their duty for want of sound judgment, she will continually illustrate by means of the subject in hand the simple rules of conduct which should guide them in the exercise of their calling, and enforce the dangers of transgressing their professional code.

